# Temporal trends in treatment strategies and clinical outcomes among patients with advanced chronic kidney disease and ST-elevation myocardial infarctions: results from the Bremen STEMI registry

**DOI:** 10.1186/s12872-022-02573-1

**Published:** 2022-04-01

**Authors:** Johannes Schmucker, Andreas Fach, Rico Osteresch, Luis Alberto Mata Marin, Tina Retzlaff, Stephan Rühle, Daniela Garstka, Uwe Kuhlmann, Ingo Eitel, Rainer Hambrecht, Harm Wienbergen

**Affiliations:** 1 From the Bremen Institute for Heart and Circulation Research, am Klinikum Links der Weser, Senator-Weßling-Str. 1, 28277 Bremen, Germany; 2 Medical Clinic II, University Heart Center, Lübeck, Germany; 3 Medical Clinic III, Department of Nephrology and Cardiology, Klinikum Bremen Mitte, Bremen, Germany

**Keywords:** ST-elevation myocardial infarction, Chronic kidney disease, Acute kidney injury, Percutaneous coronary intervention

## Abstract

**Background:**

Although the detrimental effects of advanced chronic kidney disease (CKD) on prognosis in coronary artery disease is known, there are few data on the efficacy and safety of modern interventional therapies and medications in patients with advanced CKD, because this special patient cohort is often excluded or underrepresented in randomized trials.

**Methods:**

In the present study all patients admitted with ST-elevation myocardial infarctions (STEMI) from the region of Bremen/Germany treated between 2006 and 2019 were analyzed. Advanced CKD was defined as glomerular filtration rate < 45 ml/min.

**Results:**

Of 9605 STEMI-patients, 1018 (10.6%) had advanced CKD with a serum creatinine of 2.22 ± 4.2 mg/dl at admission and with lower rates of primary percutaneous coronary intervention (pPCI) (84.1 vs. 94.1%, *p* < 0.01) and higher all-cause-mortality (44.4 vs. 3.6%, *p* < 0.01). Over time, advanced CKD-patients were more likely to be treated with pPCI (2015–2019: 90.3% vs. 2006–2010:75.8%, *p* < 0.01) and with ticagrelor/prasugrel (59.6% vs. 1.7%, *p* < 0.01) and drug eluting stents (90.7% vs. 1.3%, *p* < 0.01). During the study period a decline in adverse ischemic events (OR 0.3, 95% CI 0.1–0.7) and an increase in bleedings (OR 2.2, 95% CI 1.3–3.8) within 1 year after the index event could be observed in patients with advanced CKD while 1-year-mortality (OR 1.0, 95% CI 0.7–1.4) and rates of acute kidney injury (OR 1.2, 95% CI 0.8–1.7) did not change in a multivariate model. Both, ticagrelor/prasugrel (OR 0.48, 95% CI 0.2–0.98) and DES (OR 0.38, 95% CI 0.2–0.8) were associated with a decrease in ischemic events at 1 year.

**Conclusions:**

During the observed time period STEMI-patients with advanced CKD were more likely to be treated with primary PCI, ticagrelor or prasugrel and DE-stents. These changes probably have contributed to the decline in ischemic events and the increase in bleedings within 1 year after STEMI while overall mortality at 1-year remained unchanged for this high-risk patient group.

## Introduction

Chronic kidney disease (CKD) is an independent risk factor for developing coronary artery disease (CAD) and cardiovascular disease (CVD) so that in patients with a glomerular filtration rate (GFR) < 60 ml/min the risk for developing CAD or CVD increases significantly [1–3]. Once CAD or CVD are present in CKD, they become the leading causes of mortality and morbidity [1, 4, 5]. Furthermore, in patients with acute coronary syndromes (ACS) CKD is associated with higher rates of atypical symptoms and of in-hospital complications [6]. Current guidelines of the European society of cardiology [7] for patients with ACS/STEMI do not exempt CKD-patients from common practice-recommendations, although they advise caution regarding the use of more potent antithrombotic agents since higher bleeding rates are reported in patients with CKD. Randomized clinical trials (RCT) in ACS-patients have either excluded patients with advanced CKD or their proportion was relatively small [8]. If CKD-patients were included subgroup post-hoc analyses have shown contradictory results. In summary, there is few data on CKD-patients with ACS/STEMI focusing on the efficacy and safety of emergency percutaneous coronary interventions (PCI) using drug eluting stents (DES) and more potent P2Y12-inhibitors. Aim of the present study was therefore to investigate patients with advanced stage kidney disease and STEMI with data from a real-world clinical registry. Over the last 14 years changes in treatment practices were analyzed in a homogenous cohort of STEMI-patients and efficacy and safety of new interventional strategies and medications in STEMI and its impact on all-cause-mortality, adverse ischemic events, bleedings and acute kidney injury (AKI) were assessed.

## Methods

### The Bremen STEMI-registry (BSR)

Patients with STEMI from the greater metropolitan area of Bremen in Northwest Germany (~ 1,000,000 inhabitants) which are admitted to the Bremen Heart center are since 2006 documented in the Bremen STEMI-Registry (BSR). Regional emergency services and hospitals are connected by telephone and fax with the heart center for rapid communication to enable urgent PCI. Documentation for the BSR is done with data sheets completed by the responsible interventional cardiologist and through patient records after a cardiologist has confirmed the diagnosis. Data about age, sex, concomitant diseases, severity of STEMI, acute medical or interventional treatment, as well as laboratory parameters at admission and during the hospital stay are recorded. After discharge major adverse cardiac and cerebral events as well as bleeding events are documented through follow-up examination by telephone performed after 1, 5 and 10 years. Before inclusion of patient records in the database, a written informed consent on study participation was obtained. The study was approved by the ethical committee of the Ärztekammer Bremen, Germany. Studies about methods and results from the BSR have been previously published elsewhere [9–15].

### Study population

For this study all patients with STEMI and available creatinine values admitted between January 1st 2006 and July 31th 2019 were analyzed. The initial glomerular filtration rate was calculated with the CKD-EPI-equation at admission [16].

$${\text{GFR }} = { 141 }*{\text{ min}}\left( {{\text{Scr}}/\kappa ,{1}} \right)^{\alpha } *{\text{ max}}\left( {{\text{Scr}}/\kappa ,{ 1}} \right)^{{ - {1}.{2}0{9}}} * \, 0.{993}^{{{\text{Age}}}} *{ 1}.0{18 }\left[ {\text{if female}} \right] \, *{ 1}.{159 }\left[ {\text{if black}} \right]$$

Scr is serum creatinine (mg/dl), κ is 0.7 for females and 0.9 for males, α is − 0.329 for females and − 0.411 for males, min indicates the msinimum of Scr/κ or 1, and max indicates the maximum of Scr/κ or 1.

Patients were assigned to 4 groups by stage of kidney disease [17].

**Table:** 

Stage	GFR (ml/min/1.73^2^)
G1	≥ 90
G2	60–89
G3a	45–59
G3b-G5	< 45

### Definition of STEMI

STEMI was defined as persistent angina pectoris for ≥ 20 min in conjunction with a ST segment elevation in two contigous leads of ≥ 0.25 mV in men below the age of 40 years, ≥ 0.2 mV in men over the age of 40 years, or ≥ 0.15 mV in women in leads V2–V3 and/or ≥ 0.1 mV in all other leads or new left bundle branch block (LBBB) [7].

Subacute STEMIs were defined as STEMIs with > 12 h between first symptoms and first medical contact and/or signs of a subacute myocardial infarction in the ECG at admission.

### Surrogate parameters of severity of STEMI

Peak creatine kinase (CK) was assessed to estimate the severity of myocardial infarction. CK was routinely measured every 12 h in STEMI-patients for the first 48 h, afterwards every 24 h until hospital dismissal or transfer to a local hospital.

### Acute kidney injury

For assessment of acute kidney injury (AKI) only patients with a hospital stay of ≥ 48 h in the PCI-center were included since follow-up-controls of creatinine were needed to evaluate a possible worsening of renal function during the hospital stay. Acute kidney injury was defined with the KDIGO-AKI criterita (2012) and defined to be stage ≥ 1 with an elevation of creatinine to ≥ 1.5 of baseline or an absolute increase of ≥ 0.3 mg/dl [18].

### Bleeding criteria

For evaluation of bleeding events the Thrombolysis in Myocardial Infarction (TIMI) bleeding criteria were used to assess inhospital bleeding events [19] and stratified in TIMI minimal (< 3 g/dL decrease in haemoglobin concentration or < 9% decrease in haematocrit), TIMI minor (hemoglobin drop of 3 to < 5 g/dL or ≥ 10% decrease) and TIMI major (any intracranial bleeding or drop in hemoglobin of ≥ 5 g/dL or a ≥ 15% absolute decrease in haematocrit). A bleeding event after hospital discharge was defined as a serious bleeding event requiring medical attention.

### Definition of outcomes

In-hospital outcomes were evaluated at discharge or at time of patient-transfer to a local hospital. 30 day, 1-year- and 5-year-follow-up-outcomes were evaluated in a telephone interview. To estimate efficacy the primary ischemic endpoint was defined as a combination of in-stent-thrombosis, myocardial reinfarction and repeat target lesion revascularizations (TLR) within 1 year. Overall mortality was assessed at 1 and 5 years. The combined bleeding endpoint was defined as a combination of in-hospital bleedings (TIMI minor and major) and any significant bleeding event after hospital discharge.

### Statistical analysis

All patients admitted with STEMI to the Bremen Heart Center between January 1st 2006 and July 31th 2019 were initially assessed. For calculation of 5-year mortality rates only patients admitted between January 1st 2006 and July 31th 2015 were analyzed. Baseline characteristics of patients were described by mean values and standard deviations (SD) or standard error of mean (SEM) for continuous variables. Absolute numbers and percentages were reported for categorical variables. Univariate comparison was done with Mann–Whitney-U-tests for continuous variables (since no normal distribution was found) and for categorical variables with the extended Mantel Haenszel Chi Square test for linear trend. For the multivariate comparison a logistic regression analysis was used. To calculate changes in outcomes over time a comparative analysis vs. baseline (2006–2010) was calculated with outcomes as the dependent and time period, age, gender, diabetes and cardiogenic shock as the independent variables. To calculate the efficacy and safety of DES and prasugrel/ ticagrelor in patients with advanced CKD, a multivariate model was established, with the outcomes as the dependent variable and DES, prasugrel/ ticagrelor, age, gender, diabetes mellitus, anterior STEMI, cardiogenic shock (CS), multi-vessel disease, and peak CK as the independent variables. All calculations were done with SAS, SAS-Institute, Inc 2018.

## Results

### Study population

Between January 2006 and July 2019 9605 patients with STEMI were admitted to the Bremen heart center, documented in the Bremen STEMI-registry and entered analysis. Of these patients 3370 (35%) showed no significant reduction in GFR at admission (GFR ≥ 90 ml/min/1.73 m^2^, G1), 4005 (42%) had a mild reduction (GFR 60–89 ml/min/1.73 m^2^, G2), while in 1212 (13%) a mild to moderate reduction (GFR 45–59 ml/min/1.73 m^2^, G3a) was observed. In 1018 (11%) a moderate to severe reduction in GFR at admission was evident (GFR < 45 ml/min/1.73 m^2^, G3b-G5). They were defined as the study group with an average serum creatinine at admission of 2.22 ± 4.2 mg/dl and an estimated mean GFR of 32.3 ± 10 ml/min/1.73 m^2^. 5 of the 1018 (0.5%) in this group were on prior hemodialysis or renal replacement therapy.

### Comparison of patients by CKD-stage

Patients with advanced CKD were on average 21 years older than STEMI-patients with no CKD, were more likely to be female and to have diabetes and arterial hypertension while rates of active smoking were less. Rates of 3-vessel-disease in patients with advanced CKD were more than double compared to patients with no CKD and their rates of STEMI complicated by CS were elevated more than fourfold (Table 1). There was a trend toward use of higher CM-doses in patients with advanced CKD. Patients with advanced CKD were less likely to be treated with DES and the more potent PY12-inhibitors ticagrelor or prasugrel than patients with no relevant CKD (Table 1). With regard to in-hospital-adverse events, patients with advanced CKD showed higher rates of in-hospital-resuscitations and in-hospital bleeding events. Furthermore, in-hospital-mortality was considerably higher in patients with advanced CKD. AKI occurred at a more than fourfold higher rate in patients with advanced CKD compared to patients with no CKD. Of patients with CKD stage G3b-G5 5% required temporal and 2.5% a permanent renal replacement therapy during the hospital stay at the PCI center (Table 2).

**Table 1:** Baseline characteristics by CKD-stage

	CKD stage				
	G1 (n = 3370)	G2 (n = 4005)	G3a (n = 1212)	G3b-G5 (n = 1018)	Significance
Age (years ± SD)	54.9 ± 10	66.1 ± 12	71,8 ± 11	75.9 ± 11	< 0.01
Women (%)	19.5	26.4	38.6	46.4	< 0.01
Body mass index (BMI) (kg/m^2^ ± SD)	27.7 ± 5	27.5 ± 5	27.5 ± 5	27.2 ± 5	0.01
Diabetes (%)	16.2	17.6	24.9	29.8	< 0.01
Current smokers (%)	63.8	35.4	22.7	16.2	< 0.01
Arterial hypertension (%)	50.9	63.8	71.7	72.1	< 0.01
Medical history					
PCI (%)	9.4	12.8	15.5	14.8	< 0.01
Acute myocardial infarction (%)	9.1	12.7	16.4	17.5	< 0.01
CABG (%)	1.4	2.7	4.4	4.9	< 0.01
Stroke/TIA (%)	3.0	5.9	8.4	10.1	< 0.01
Treatment strategy					
Primary PCI (%)	94.1	89.8	86.3	84.1	< 0.01
Urgent/early elect. CABG (%)	3.0	5.3	7.4	7.5	< 0.01
Conservative (%)	2.8	4.9	6.3	8.5	< 0.01
Systemic thrombolysis with (rescue) PCI (%)	1.1	2.1	4.5	4.2	< 0.01
Systemic thrombolysis (stand alone) (%)	0	0.15	0	0	–
Coronary vessels diseased					
1 (%)	45.6	36.2	29.5	21.7	< 0.01
2 (%)	30.6	31.2	32.4	29.8	0.05
3 (%)	23.7	32.6	38.1	48.5	< 0.01
Subacute STEMI (%)	11.1	12.6	12.4	13.8	0.02
Cardiogenic shock (%)	6.4	10.4	23.7	30.9	< 0.01
TIMI 0/1 flow after PCI (%)	2.6	4.5	7.1	10.6	< 0.01
Peak CK > 2000 U/l (%)	31.9	31.4	30.5	28.8	0.07
Creatine at admission (mg/dl ± SD)	0.75 ± 0.13	0.97 ± 0.15	1.23 ± 0.18	2.22 ± 4.15	< 0.01
eGFR at admission (ml/min ± SD)	101 ± 8.4	76.2 ± 8.6	53.2 ± 4.2	32.3 ± 10.0	< 0.01
Contrast media used (ml ± SD)	143.9 ± 61	146.5 ± 61	144.1 ± 63	144.6 ± 71	0.3
Contrast media > 150 ml (%)	33.3	35.8	34.9	36.4	0.06
Stent type					
Bare metal stent (%)	36.8	46.6	50.5	45.8	< 0.01
Drug eluting stent (%)	59.3	47.5	41.1	44.1	< 0.01
No stent (POBA) (%)	3.8	5.8	8.4	10.0	< 0.01
Medication at discharge					
ASS (%)	98.9	97.1	92.6	89.4	< 0.01
Clopidogrel (%)	25.6	46.8	57.1	56.3	< 0.01
Ticagrelor or prasugrel (%)	70.9	46.3	31.8	31.7	< 0.01
Triple therapy (%)	8.4	12.2	16.2	12.6	< 0.01
Statin (%)	95.5	89.2	78.3	68.1	< 0.01
ACE-inhibitor/ATR (%)	90.3	82.1	70.4	55.8	< 0.01
Betablocker (%)	77.8	80.4	75.7	64.5	< 0.01

**Table 2:** In hospital and 1-year outcomes by CKD-stage

	CKD stage				
	G1 (n = 3370)	G2 (n = 4005)	G3a (n = 1212)	G3b-G5 (n = 1018)	Significance
In hospital events					
Resuscitations (%)	1.9	3.3	6.9	9.3	< 0.01
Cerebrovascular events/TIA (%)	0.2	0.6	1.1	0.8	< 0.01
TIMI minimal bleedings (%)	6.2	7.7	11.4	13.8	< 0.01
TIMI minor bleedings (%)	0.9	1.5	2.3	2.6	< 0.01
TIMI major bleedings (%)	0.7	1.5	2.9	3.5	< 0.01
Overall in-hospital mortality (%)	1.3	4.9	14.9	28.4	< 0.01
Acute kidney injury KDIGO stg. 1 + (%)	8.4	12.8	28.4	41.9	< 0.01
AKI requiring temporal renal replacement therapy (%)	0.4	0.9	3.1	5.0	< 0.01
AKI requiring permanent renal replacement therapy (%)	0	0.04	0	2.5	< 0.01
Adverse events at 1 year					
Stent-thrombosis (ST) (%)	1.3	1.5	1.9	1.7	0.19
Myocardial re-infarction (AMI) (%)	3.7	4.3	4.3	5.5	0.03
Repeat target lesion revascularisation (TLR) (%)	2.1	2.3	1.6	1.3	0.41
Cumulative ischemic events (ST + AMI + TLR) (%)	5.1	6.3	5.8	6.5	0.12
Cerebrovascular events/TIA (%)	0.9	1.8	1.9	3.0	< 0.01
Overall mortality (%)	3.6	11.2	27.9	44.4	< 0.01
Bleeding events after hospital discharge (%)	2.2	2.7	3.4	4.1	< 0.01
Cumulative bleeding events (TIMI min + major or any bleeding after disch.) (%)	3.7	5.4	8.2	9.4	< 0.01

At 1 year, patients with advanced CKD were more likely to suffer recurrent myocardial infarctions, while rates of in-stent-thrombosis or a repeat target lesion revascularization (TLR) did not differ across CKD-stages. Patient with advanced CKD showed higher rates of 1-year-mortality, cerebrovascular events and bleeding events after hospital discharge compared to patients with no CKD (Table 2).

To rule out confounding by the higher prevalence rates of cardiogenic shock (CS) in patients with advanced CKD, event rates were separately assessed for a subgroup of only patients with CS on admission. This subgroup-analysis showed that while overall rates were higher, the association between CKD-stage and elevation in event rates remained evident: In-hospital-mortality: G1: 9.2%, G2: 21.4%, G3a: 39.7%, G3b-G5: 60%, *p* < 0.01; 1-year-mortality: G1: 12.4%, G2: 32.7%, G3a: 55.8%, G3b-G5: 78.9%; *p* < 0.01; AKI: G1: 18.1%, G2: 32.7%, G3a: 50.3%, G3b-G5: 60.2%, *p* < 0.01.

### Temporal changes in baseline characteristics and interventional details in patients with advanced CKD

Over time the mean age of patients with advanced CKD increased by 2.1 years while the proportion of women did not change. Furthermore, obesity decreased slightly and active smoking declined. Over time patients with advanced CKD were more likely to be treated with PCI, while rates of emergency or urgent coronary artery bypass grafting (CABG) or conservative treatment strategies in STEMI-patients more than halved (Table 3). Neither severity of CAD nor rates of CS or rates of more extensive STEMIs (estimated by peak CK) changed significantly during the study period. However, the preferred stent type changed over time. After 2015 90.7% patients with advanced CKD received a DES and at the same time a more than 40% increase of the mean number of stents implanted could be observed. Furthermore, an increase in the use of ticagrelor and prasugrel at discharge could be seen in accordance with changes in guidelines recommendations and the recommended duration of a dual antiplatelet therapy (DAPT) increased on average by more than 8 months (Table 3).

**Table 3:** Temporal trends of patient characteristics’ in patients with advanced CKD (G3b-G5)

	Time period			
	T1 2006–2010 (n = 407)	T2 2011–2015 (n = 313)	T3 2016–2019 (n = 298)	Significance
Age (years ± SD)	74.5 ± 10	76.8 ± 12	76.6 ± 11	0.012
Women (%)	44.7	53.2	42.6	0.72
Body mass index (BMI) (kg/m^2^ ± SD)	27.5 ± 5	27.1 ± 5	26.9 ± 5	0.09
Diabetes (%)	32.0	29.0	27.4	0.15
Current smokers (%)	19.2	16.2	11.8	< 0.01
Arterial hypertension (%)	72.6	73.6	70.0	0.46
Treatment strategy				
Primary PCI (%)	75.8	88.8	90.3	< 0.01
Urgent/early elect. CABG (%)	10.4	6.1	5.1	< 0.01
Conservative (%)	13.8	5.1	4.7	< 0.01
Coronary vessels diseased				
1 (%)	19.3	26.7	19.5	0.79
2 (%)	32.7	29.1	26.7	0.09
3 (%)	48.1	44.1	53.8	0.2
Cardiogenic shock (%)	29.9	28.8	34.6	0.24
TIMI 0/1 flow after PCI (%)	11.1	10.8	9.8	0.56
Peak CK > 2000 U/l (%)	26.6	30.1	30.3	0.29
Contrast media used (ml ± SD)	132 ± 62	153 ± 76	152 ± 73	< 0.01
Contrast media > 150 ml (%)	27.7	43.0	41.6	< 0.01
Stent type in PCI cohort				
Bare metal stent (%)	88.3	42.8	0.3	< 0.01
Drug eluting stent (%)	1.3	46.4	90.7	< 0.01
No stent (POBA) (%)	10.4	10.7	8.9	0.52
No. of stents impl. (n ± SD)	1.3 ± 0.6	1.5 ± 0.8	1.8 ± 1.0	< 0.01
Medication at discharge				
ASS (%)	91.9	89.1	86.5	< 0.02
Clopidogrel (%)	83.0	48.3	28.2	< 0.01
Ticagrelor or prasugrel(%)	1.7	44.1	59.6	< 0.01
Triple therapy (%)	16.1	12.1	8.8	< 0.01
DAPT-duration (months ± SD)	2.5 ± 3	7.9 ± 5	10.9 ± 4	< 0.01
DAPT-duration > 6 months (%)	8.9	58.2	89.2	< 0.01
Statin (%)	71.4	66.1	65.6	0.11
ACE-inhibitor/ATR (%)	55.6	56.7	55.1	0.88
Betablocker (%)	70.4	61.9	59.6	< 0.01

### Temporal changes in short- and long-term outcome in patients with advanced CKD

Over time an increase in-hospital resuscitations could be observed, while rates of TIA/cerebrovascular events and overall mortality did not change. Rates of TIMI minimal bleedings increased as well as TIMI major bleedings. Despite on average higher doses of contrast media the incidence of AKI following contrast media exposure did not change significantly (Tables 3, 4). At 1 year a significant decrease of in-stent-thrombosis was evident, as well as a decrease in repeat TLR. Rates of myocardial re-infarction more than halved during the study period. In contrast rates of bleeding events after hospital discharge increased significantly, while 1-year and 5-year overall mortality did not change (Table 4).

**Table 4:** Temporal trends in in-hospital and 1-year-outcomes in patients with advanced CKD (G3b-G5)

	Time period			
	T1 2006–2010 (n = 407)	T2 2011–2015 (n = 313)	T3 2016–2019 (n = 298)	Significance
In hospital events				
Resuscitations (%)	6.9	8.9	13.1	< 0.01
Cerebrovascular events/TIA (%)	0.5	0.9	1.0	0.58
TIMI minimal bleedings (%)	10.7	10.8	21.1	< 0.01
TIMI minor bleedings (%)	3.2	1.6	2.7	0.49
TIMI major bleedings (%)	2.2	1.9	6.7	< 0.01
Overall mortality (%)	26.6	29.3	30.6	0.18
Acute kidney injury (KDIGO stage 1 + (%))	38.3	44.8	41.6	0.47
Adverse events at 1 year				
In-stent-thrombosis (ST) (%)	3.0	1.3	1.0	0.047
Myocardial re-infarction (AMI) (%)	7.1	5.4	3.1	< 0.01
Repeat target lesion revascularisation (TLR) (%)	3.3	0.3	1.5	0.04
Cumulative isch. events (ST + AMI + TLR) (%)	10.8	5.7	3.1	< 0.01
Cerebrovascular events/TIA (%)	3.0	3.3	2.7	0.74
Overall mortality (%)	46.9	47.7	51.5	0.29
Bleeding events after hospital discharge (%)	2.0	6.0	5.5	0.044
Cumulative bleeding events (TIMI minor + major + any bleeding event after discharge.) (%)	6.9	8.9	13.4	< 0.01
Adverse events at 5 years				
Overall mortality (%)	67.2	66.4	–	0.72

**Fig. 1:**
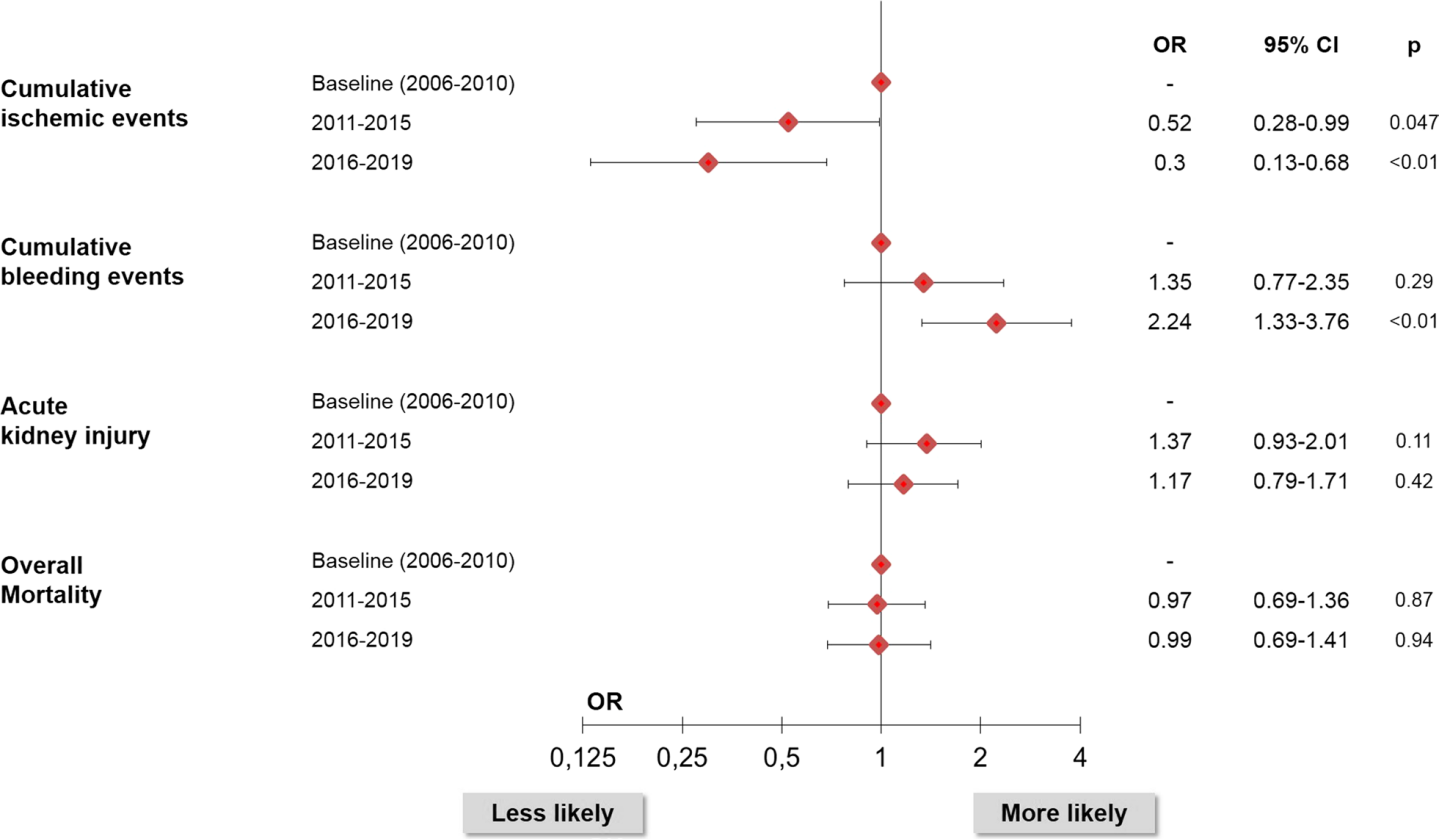
Multivariate analysis of temporal changes of inhospital and 1-year-outcomes in STEMI-patients with advanced CKD. Outcome models comparing time periods with 2006–2010 as baseline. OR and 95% CIs calculated in a multivariate model adjusted for age, gender, diabetes and cardiogenic shock. Cumulative ischemic events, bleeding-events and overall-mortality compared at 1-year, AKI with in-hospital-rates

The results of the univariate analysis over time could largely be confirmed in a multivariate model and was calculated for in-hospital or 1-year-event rates adjusted for possible confounders: A significant decrease in cumulative ischemic events at 1 year was evident: 2011–2015 OR 0.52, (95% CI 0.28–0.99); 2016–2019 OR 0.3, (95% CI 0.13–0.68); 2006–2010 = baseline, while rates of cumulative bleedings within 1 year after the index event increased during the observation period: 2011–2015 OR 1.35, (95% CI 0.77–2.35), 2016–2019: OR 2.24, (95% CI 1.33–3.76); 2006–2010 = baseline. Again, overall mortality at 1-year did not change nor did rates of AKI (Fig. 1).

### Impact of more potent P2Y12-inhbitors and modern stents on outcome in patients with advanced CKD

To analyze the impact of the more potent P2Y12-inhibitors prasugrel and ticagrelor (compared to clopidogrel) and DES (compared to BMS) in CKD-patients on outcome, event rates (in-hospital and at 1-year) were first compared in a univariate model. Both prasugrel/ticagrelor and DES were associated with a significant decrease in cumulative ischemic events within 1 year after the index event (p/t vs. clopidogrel: 4.3 vs. 8.7%, *p* = 0.037, DES vs. BMS: 3.8 vs. 10.2%, *p* < 0.01), while cumulative bleeding rates were only higher with DES (12.7 vs. 7.5%, *p* = 0.018) and did not differ between type of P2Y12-inhibitor (p/t vs. clopidogrel: 9.8 vs. 10.2%, n.s.). Overall mortality at 1-year in patients with CKD was neither effected by type of P2Y12-inhibitor nor stent type.

**Fig. 2:**
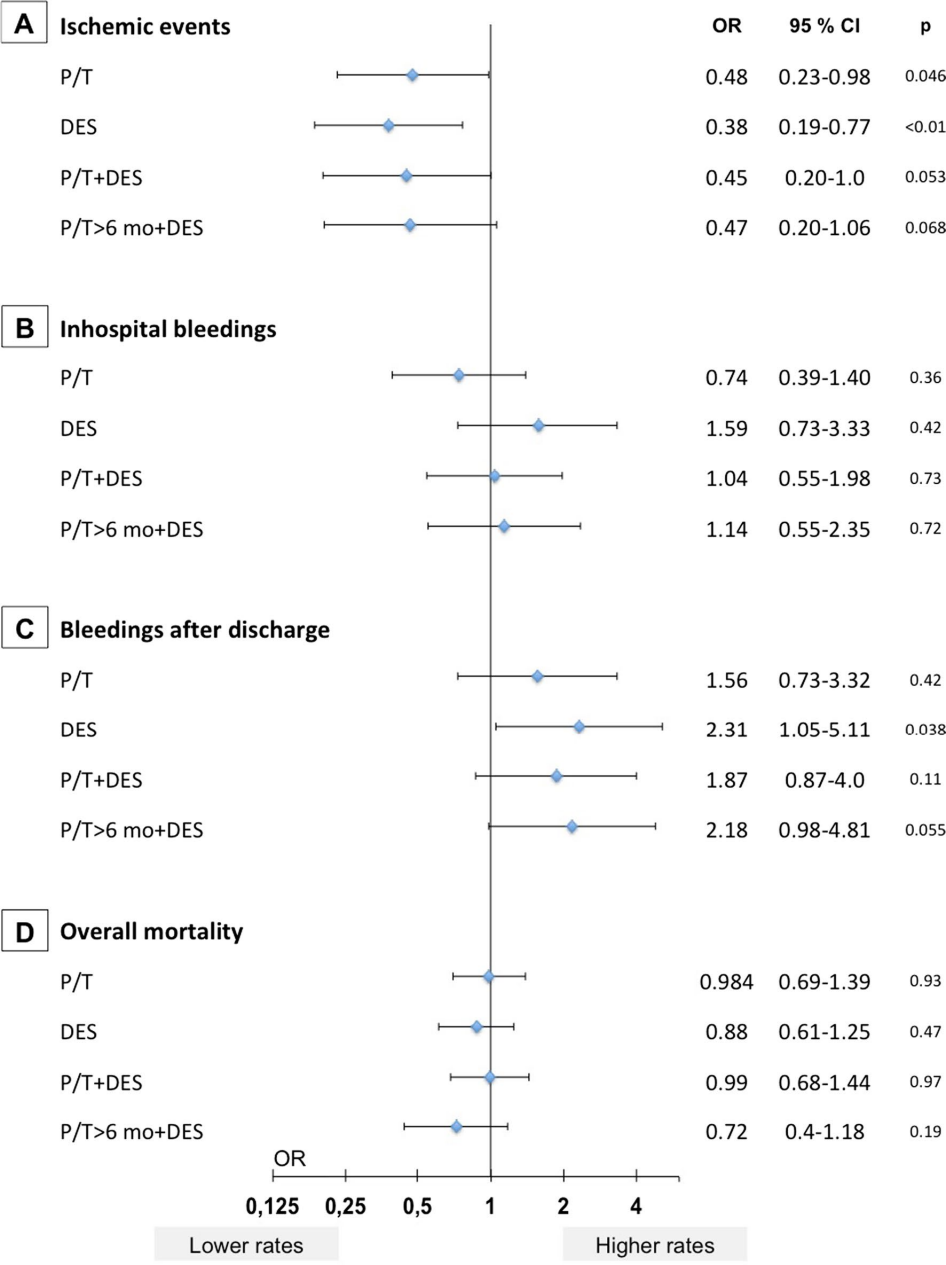
Multivariate model on the impact of prasugrel/ticagrelor, DES and duration of DAPT on inhospital and 1-year-outcomes of STEMI-patients with advanced CKD. Outcome models calculated for cumulative ischemic events at 1-year (ST + AMI + TLR) (**A**), inhospital bleedings (**B**), bleedings after discharge (within 1 year after the index event) (**C**) and overall mortality at 1-year (**D**). Multivariate model adjusted for age, gender, diabetes mellitus, anterior STEMI, cardiogenic shock, multivessel disease, and peak CK; P/T indicates prasugrel or ticagrelor (compared to clopidogrel); DES indicates drug-eluting stents (compared to BMS); > 6 mo indicates recommended dual antiplatelet therapy (DAPT) for longer than 6 months

When analyzing effects of modern therapies in a multivariate model a stepwise approach was chosen. In a model adjusted for confounders both prasugrel/ticagrelor (compared to clopidogrel) and DES (compared to BMS) were individually associated with lower ischemic event rates at 1-year and this advantage remained when both therapies were combined (Fig. 2A). In-hospital bleedings were not significantly affected by the use of prasugrel, ticagrelor or the use of DES or a longer duration of DAPT-therapy (Fig. 2B). In contrast, treatment with prasugrel or ticagrelor combined with the use of a DES and a longer duration of DAPT was associated with a significant increase in bleeding events after hospital discharge (Fig. 2C). Overall mortality at 1 year again was not influenced by modern P2Y12-inhibitors, DES or the combination of both (Fig. 2D).

## Discussion

The present analysis from an all-comers clinical registry demonstrates that during the last 14 years STEMI-patients with advanced CKD, which constituted 11% of the total cohort, were more likely to be treated with emergency PCI and are in their majority now treated with DES and the more potent P2Y12-inhibitors ticagrelor and prasugrel. Possibly also due to these changes, adverse ischemic event rates after STEMI more than halved during this time, however at the cost of an increase in bleedings. Overall mortality remained high for this high-risk-population and was for now not affected by the decrease in ischemic events.

### Pathogenesis of CKD and its role in CAD and CVD

Multiple studies have shown, that with declining renal function the risk to develop CVD or CAD increases linearly [1–3]. Previous studies have demonstrated, that this negative impact is partly triggered by the high prevalence rates of arterial hypertension and diabetes mellitus, which have detrimental effects on overall prognosis and vascular complications [3]. It remains uncertain whether a primary renal disease besides the classical risk factors contributes to the development of CVD in CKD and whether other pathological mechanisms could also play a role. A study comparing the cardiovascular risk in CKD with and without coexisting glomerulonephritis (GN) found that the presence of GN by itself did not increase the likelihood of developing CAD/CVD [20]. The importance of diabetes and hypertension in CKD could indirectly be confirmed in our results, since rates of diabetes or hypertension were considerably higher in patients with advanced CKD, while rates of active smoking were considerably lower with smoking-rates of 16.2% in advanced CKD compared to 63.8% in patients with no CKD.

### Impact of advanced CKD on outcome in CAD or ACS

CKD, especially in more advanced stages, is known to be associated with worse short and long-term prognosis in patients with acute myocardial infarctions [3, 5] for different reasons. First, in the preclinical phase of AMI, patients with advanced CKD are more likely to present with atypical symptoms [6, 21], which may delay diagnosis. Second, patients with advanced CKD are more likely to show more complex CAD with higher degree of coronary calcification, which can be more challenging for an intervention. And third, advanced CKD in ACS/STEMI-patients may cause restraint in the interventional cardiologist since nephropathy after contrast media exposure is more common in patients with known CKD [14, 22]. These observations could mostly be confirmed in the present data: Advanced CKD was associated with a marked increase of in-hospital and 1-year-mortality and was associated with higher rates of in-hospital complications and higher degree of coronary calcification.

While the importance of CKD on the probability of AKI following contrast media exposure in STEMI-patients undergoing emergency PCI has been shown in prior studies a recently published analysis from our center showed, that in STEMI the main contributor to AKI is more likely the STEMI itself and its hemodynamic sequelae and to a lesser degree a CM-induced renal damage [14]. This observation was indirectly confirmed in the present study: While over time STEMI-patients with advanced CKD received on average higher CM-doses since emergency PCI was more often attempted and an increasing number of stents was implanted, these changes were not accompanied with a significant increase in AKI-rates.

### Interventional strategies in patients with advanced CKD and stable angina or ACS

Previous studies on the possible benefit of early revascularisation strategies in patients with ACS/NSTEMI and CKD have generally shown a benefit for early revascularisation despite a reduced renal function [23, 24]. Studies focusing on STEMI-patients with advanced CKD are scarce. A registry analysis from Malaysia showed that STEMI-patients with advanced CKD had a worse prognosis and higher vascular complication-rates than patients with no CKD [25]. However, primary PCI-rates were comparatively low at 21% with high rescue PCI-rates after failed systemic fibrinolytic therapy at 70%.

The present study confirms prior findings: Prognosis for STEMI-patients with advanced CKD is far worse, with a marked increase in short- and long-term mortality, higher bleeding rates and a nearly fivefold higher risk of AKI. However, especially compared to data from Malaysia, primary PCI-rates in CKD-patients with STEMI in the present study were high and increased even further over time and in recent years are nearly comparable to STEMI-patients without preexisting CKD.

### Efficacy of modern stent generations and choice of P2Y12-inhibitors in patients with advanced CKD

Current literature provides few studies, focusing on the possible benefit of modern DES in patients with advanced CKD. In one prospective registry study published in 2010, DES were associated with lower risk of all-cause-death and major adverse cardiovascular event-rates (MACE) compared to BMS [26]. Similarly, a meta-analysis, published in 2018 showed that DES in patients with CKD were superior to BMS resulting in lower adverse ischemic event rates and a 18% lower all-cause mortality [27]. The study authors’ argue that the advantage is probably explained by a lower risk of stent-thrombosis and the need for TVR/TLR in CKD-patients treated with a DES. Our findings in STEMI confirm these observations only in part. While the use of DES was associated with lower adverse ischemic event rates (ST + AMI + TLR) they were not associated with lower all-cause-mortality-rates. This might be due to the characteristics of the present study with high rates of concomitant cardiogenic shock in STEMI-patients with advanced CKD, which in itself is a major predictor for mortality. Furthermore, in the meta-analysis by Crimi et al. [27], safety events like bleedings or AKI were not assessed and only a minority of patients had STEMI, which also may explain the different results.

Studies focusing on type of P2Y12-inhibitor prior studies have shown contradictory results: While in the CLARITY-TIMI-28 trial [28], which compared clopidogrel to placebo, showed that patients with CKD did not benefit from clopidogrel, a subgroup-analysis form the TRITON-TIMI-38-trial [29], which compared prasugrel to clopidogrel showed, that the risk reduction in patients with no CKD was more pronounced at 20%, while patients with CKD (GFR < 60 ml/min) showed a reduction of 14%. Conversely, the PLATO-trial [30], comparing ticagrelor to clopidogrel showed, that the risk–reduction was more pronounced in patients with CKD with a HR 0.77, compared to a HR of 0.92 in patients with preserved renal function.

In general, randomized trials differ from “all-comers” registry data, because old and comorbid patients often do not participate in RCTs. It is therefore crucial to demonstrate efficacy and safety of the new therapeutic strategies in “real world” data. We show that prasugrel and ticagrelor and DES are associated with lower ischemic events and a trend towards more bleedings after 1 year. Thus, the new P2Y12-inhibitors and DES are safe and efficient in the clinical management of patients with STEMI and advanced CKD, although moderately increased bleeding rates can be observed.

## Conclusions

This study demonstrated that in recent years, patients with advanced kidney disease and STEMI were more likely to undergo emergency PCI with drug eluting stents and the majority were treated with more potent P2Y12-inhbitors (ticagrelor or prasugrel). This probably contributed to the decrease in adverse ischemic events after STEMI, which more than halved during the study period. On the contrary, an increase in bleeding events could be observed, while overall mortality at 1 and 5 years remained high and unchanged.

This “all-comers” registry therefore shows the efficacy and safety of new therapeutic strategies in STEMI-patients with advanced CKD. The study furthermore demonstrates that patients with impaired renal function are a special cohort in the management of STEMIs, with an increased risk of total adverse events compared to patients without CKD.

## Limitations

This study is limited by the definition of kidney disease as it was estimated with the calculated initial renal function, which might not in all patients truly reflect renal function before the index event and may lead to indication bias. Furthermore, this is an observational registry study and any analysis assessing causal relationships may be affected by residual confounding.

## Data Availability

The datasets generated and/or analysed during the current study are not publicly available due to their sensitive nature but may be available from the corresponding author on reasonable request.

## Abbreviations

ACE: Angiotensin converting enzyme
ACS: Acute coronary syndrome
ARB: Angiotensin II receptor blocker
BMS: Bare metal stent
BSR: Bremen STEMI registry
CABG: Coronary artery bypass graft
CAD: Coronary artery disease
CVD: Cardiovascular disease
CI: Confidence interval
CK: Creatine kinase
CKD: Chronic kidney disease
CM: Contrast media
CS: Cardiogenic shock
DAPT: Dual antiplatelet therapy
DES: Drug eluting stent
GFR: Glomerular filtration rate
LBBB: Left bundle branch block
OR: Odds ratio
PCI: Percutaneous coronary intervention
RCT: Randomized controlled trial
ST: Stent thrombosis
STEMI: ST-elevation myocardial infarction
TIMI: Thrombolysis in myocardial infarction
TLR/TVR: Target lesion/target vessel revascularisation
